# Supplementary material for: Firefly-inspired bipolar information indication system actuated by white light
Source: Nat Commun. 2024 May 11;15:3999. doi: 10.1038/s41467-024-48473-0 (PMC11088637; doi:10.1038/s41467-024-48473-0)
Supplement: Supplementary file 1 — Supplementary Information [file 41467_2024_48473_MOESM1_ESM.pdf]

Supplementary Information

# Firefly-inspired Bipolar Information Indication System Actuated by White Light

*Hanwen Huang [1†], Jiamiao Yin [2†], Qianwen Zhou [3], Huateng Li [1], Junying Yang [1], Yaoben Wang [1], Ming Xu [2,4]* and Changchun Wang [1]**

[1] State Key Laboratory of Molecular Engineering of Polymers, Department of Macromolecular Science, Laboratory of Advanced Materials, Fudan University, Shanghai, 200433, China.

[2] State Key Laboratory of Molecular Engineering of Polymers, Department of Chemistry, Fudan University, Shanghai, 200433, China.

[3] Institute of Microscale Optoelectronics, Shenzhen University, Shenzhen 518060, China.

[4] School of Chemistry and Chemical Engineering, Shanghai Jiao Tong University, Shanghai 200240, China

[†] These authors contributed equally to this work.

[*] Corresponding author. E-mail address: ccwang@fudan.edu.cn (C. C. Wang); xu-ming@sjtu.edu.cn

## TABLES

**Supplementary Table 1.** DLS size, polymer dispersity index (PDI) after each step of emulsion polymerization for red, gold, green and blue photonic crystal films. Samples were prepared by 500 mL stepwise emulsion polymerization with 0.08 g (Red), 0.09 g (Gold), 0.10 g (Green) and 0.13 g (Blue) SDS in the seed preparation, respectively.

| **Sample Name** | | **Size (nm)** | **PDI** |
|---|---|---|---|
| Red | Seed | 52 | 0.056 |
| | Core | 188 | 0.033 |
| | CI | 207 | 0.039 |
| | CIS | 225 | 0.037 |
| Gold | Seed | 47 | 0.062 |
| | Core | 166 | 0.012 |
| | CI | 190 | 0.031 |
| | CIS | 204 | 0.017 |
| Green | Seed | 40 | 0.057 |
| | Core | 140 | 0.019 |
| | CI | 159 | 0.026 |
| | CIS | 186 | 0.028 |
| Blue | Seed | 32 | 0.069 |
| | Core | 113 | 0.032 |
| | CI | 121 | 0.035 |
| | CIS | 147 | 0.025 |

**Supplementary Table 2.** Absorption peak, emission peak and FWHW (full width at half maximum) of different emitters.

| **Emitter** | **Absorption peak/nm** | **Emission peak/nm** | **FWHW/nm** |
|---|---|---|---|
| Perylene | 436, 409, 388 | 450, 470, 504 | 39 |
| Bodipy | 500 | 525 | 32 |
| Eu complex | 342 | 614 | 8 |

**FIGURES**

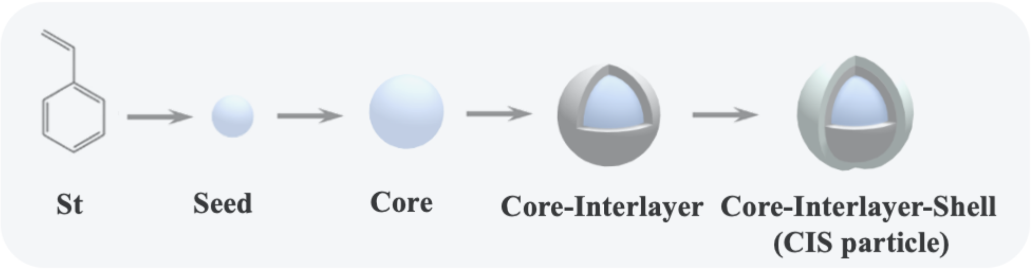


**Supplementary Figure 1. Schematic diagram.**

Schematic of stepwise emulsion polymerization to fabricate CIS particles.

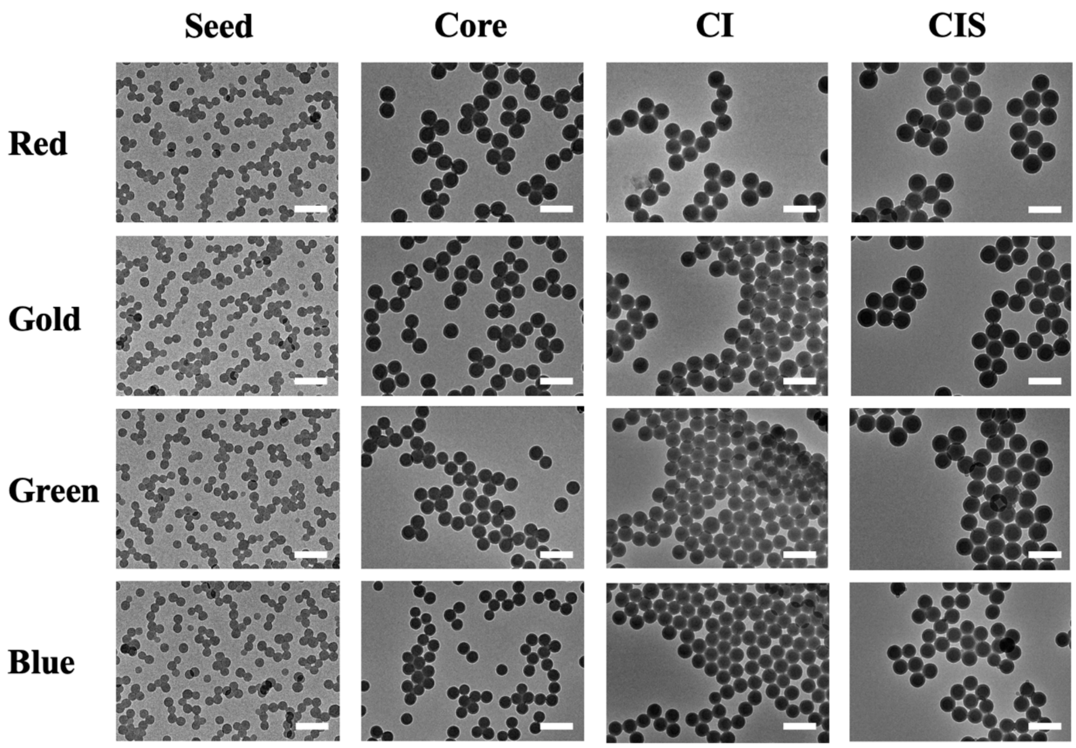


**Supplementary Figure 2. TEM images.**

TEM images of particles after each step of emulsion polymerization for red, gold, green and blue photonic crystal films. Samples were prepared by 500 mL stepwise emulsion polymerization with 0.08 g (Red), 0.09 g (Gold), 0.10 g (Green) and 0.13 g (Blue) SDS in the seed preparation, respectively. The scale bar is 350 nm.

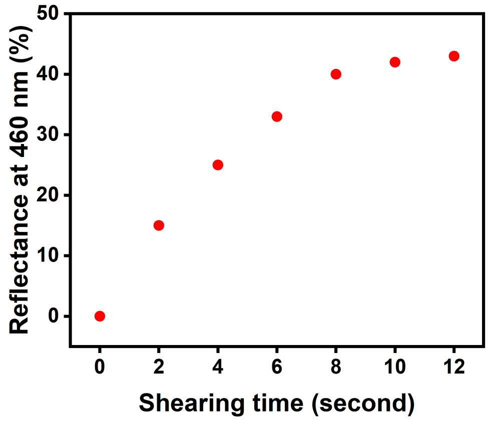


**Supplementary Figure 3. Reflectance.**

Reflectance of blue photonic crystal film undergoing different shearing time (the rotation-shearing angular velocity was 1/3 rad/s and rotation-shearing amplitude 1/6 rad with corresponding rotation-shearing frequency at 0.5 Hz).

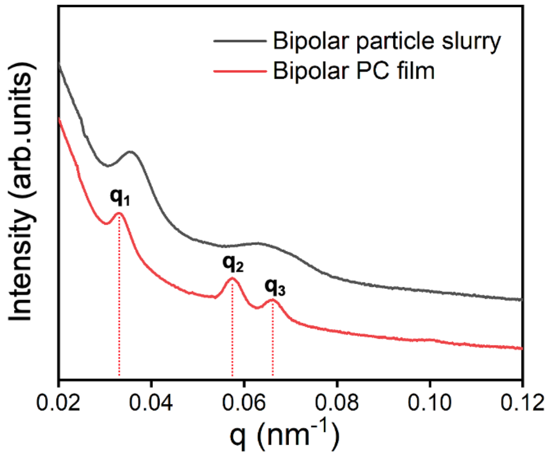


**Supplementary Figure 4. 1D USAXS.**

1D USAXS curves of bipolar particle slurry (disordered, before SIOT) and bipolar photonic crystal film (ordered, after SIOT).

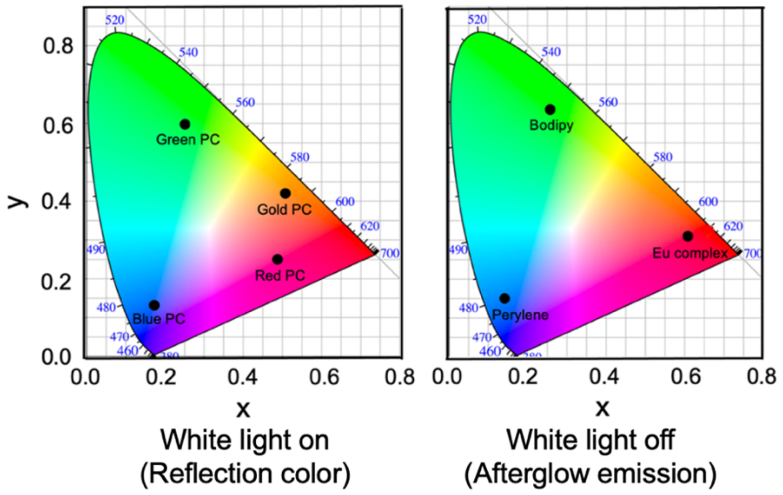


**Supplementary Figure 5. CIE diagram.**

CIE diagram of bipolar information indication system with different reflection colors and afterglow when white light is on and off, respectively.

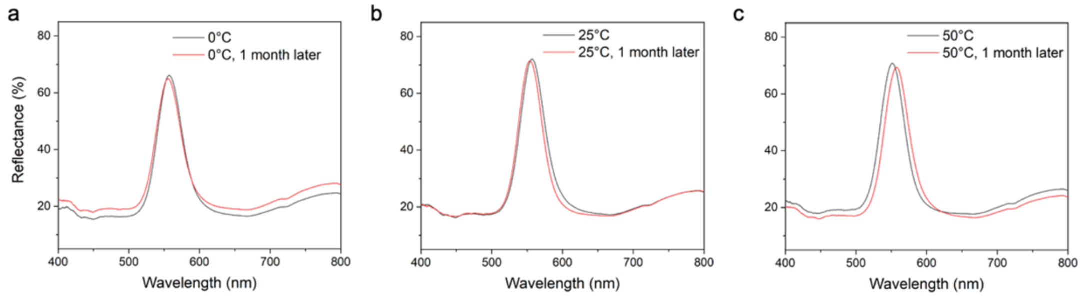


**Supplementary Figure 6. Reflectance.**

Reflectance of photonic crystal films stored under (a) 0°C, (b) 25°C and (c) 50°C for 1 month.

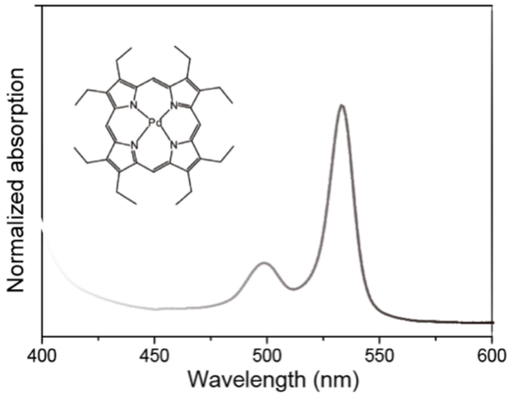


**Supplementary Figure 7. Absorbance.**

Absorbance spectrum of sensitizer PdOEP.

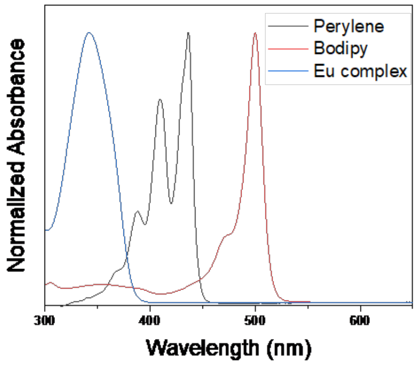


**Supplementary Figure 8. Absorbance.**

Absorbance spectra of different emitters.

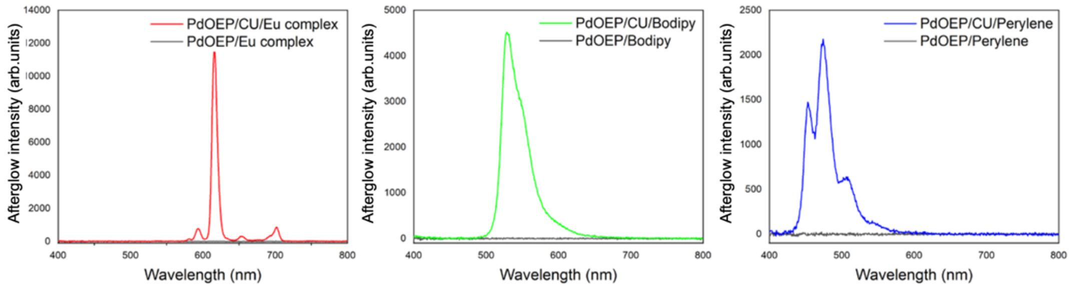


**Supplementary Figure 9. Afterglow emission spectra with and without CU.**

Afterglow emission spectra of photochemical reactions with different emitters, with and without the presence of CU. The afterglow emission spectrum was collected after being irradiated with white light for 1 min with a fixed power density of 50 mW/cm$^2$.

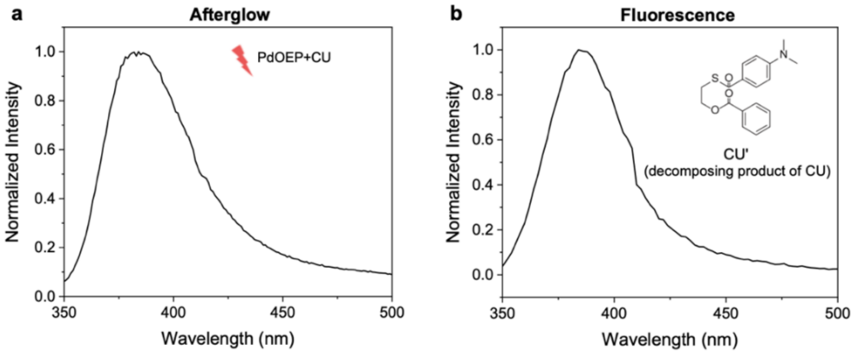


**Supplementary Figure 10. Afterglow emission spectra without emitter.**

(a) Afterglow emission spectra of CU sensitized by PdOEP without adding emitter. The afterglow emission spectrum was collected after being irradiated with white light for 1 min with a fixed power density of 50 mW/cm$^2$. (b) Photoluminescence emission spectrum of decomposing product of CU in toluene ($\lambda_{Ex}$=330 nm).

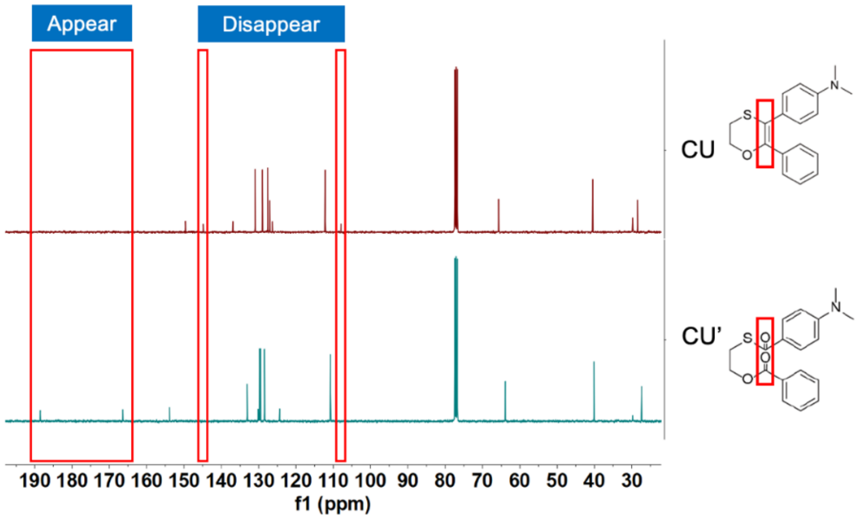


**Supplementary Figure 11. Nuclear magnetic resonance (NMR) spectra.**

$^{13}$C NMR spectra of CU and CU', with deuterated chloroform ($CDCl_3$) as solvent.

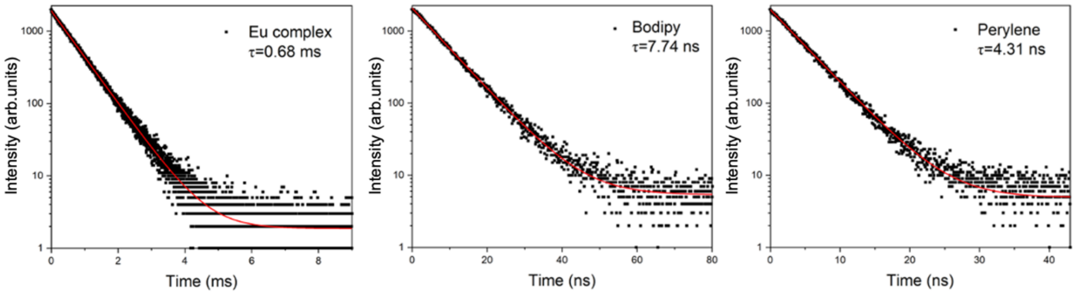


**Supplementary Figure 12. Fluorescence decays.**

Fluorescence decays and lifetime of different emitters.

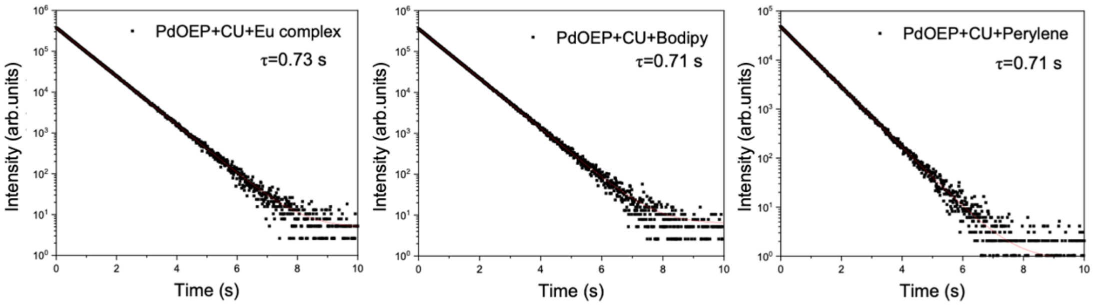


**Supplementary Figure 13. Afterglow decays.**

Afterglow decays of photochemical reaction systems with different composition of emitters and their lifetime.

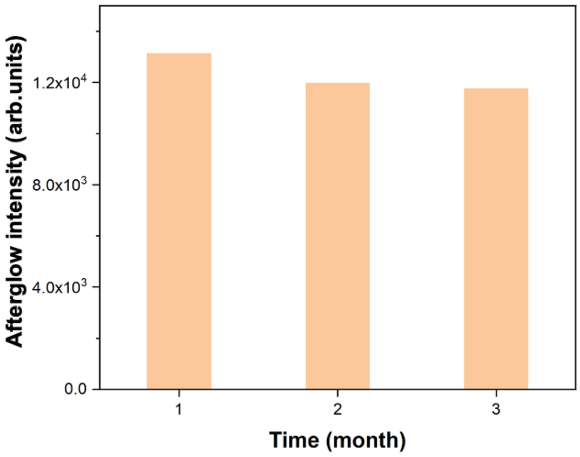


**Supplementary Figure 14. Afterglow intensity.**

Afterglow intensity of bipolar PC film at a CU concentration of 12 mM stored in darkness for different time periods.

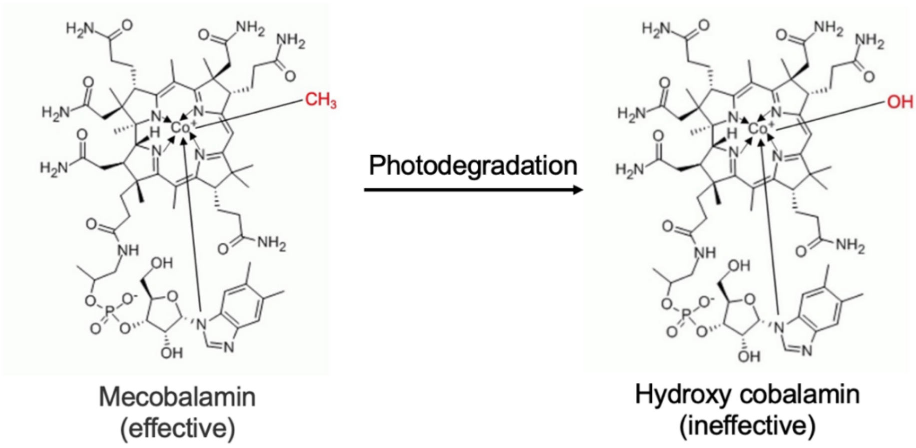


**Supplementary Figure 15. Chemical formula.**

The chemical formula of mecobalamin and hydroxy cobalamin.

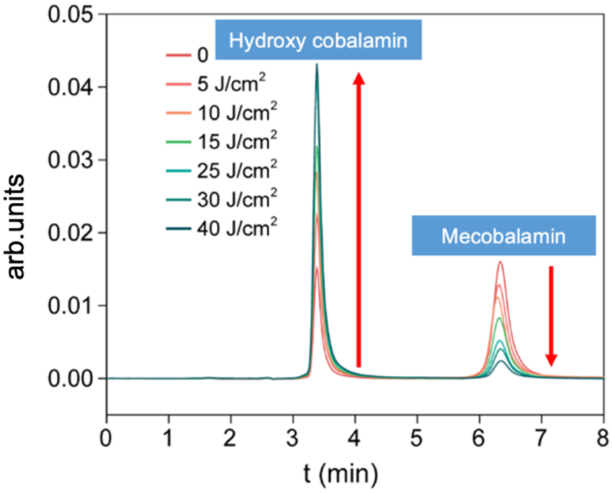


**Supplementary Figure 16. High performance liquid chromatograph (HPLC) chromatogram.**

A typical HPLC of mecobalamin under different white light dose.

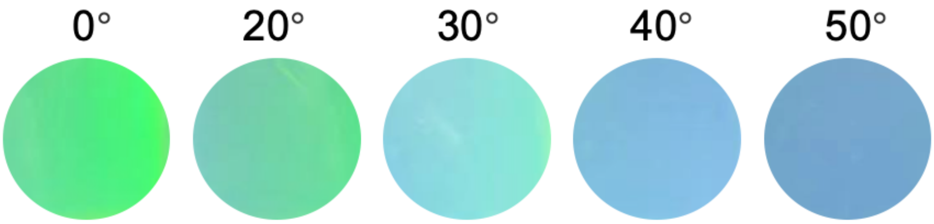


**Supplementary Figure 17. Anti-counterfeiting patterns.**

Pictures of anti-counterfeiting patterns at different viewing angles.

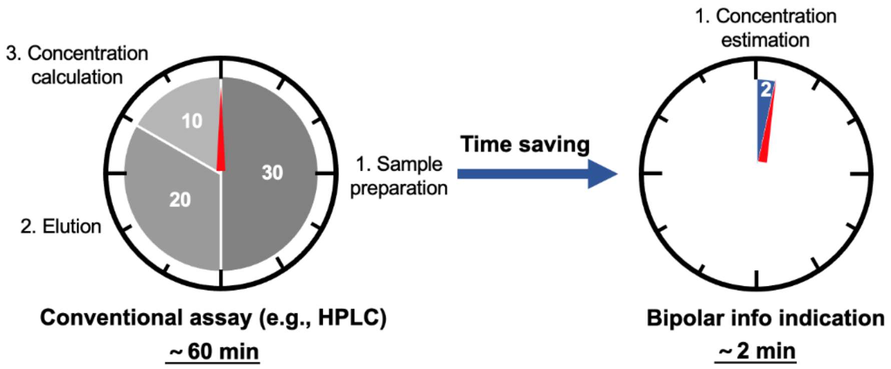


**Supplementary Figure 18. Schematic diagram.**

Schematic illustration of time required for indicating the photodegradation level of photosensitive medicine through conventional assay and bipolar information indication.
